# Long-Term Safety of Facilitated Subcutaneous Immunoglobulin 10% Treatment in US Clinical Practice in Patients with Primary Immunodeficiency Diseases: Results from a Post-Authorization Safety Study

**DOI:** 10.1007/s10875-024-01769-8

**Published:** 2024-08-19

**Authors:** Arye Rubinstein, Mohsen Mabudian, Donald McNeil, Niraj C. Patel, Richard L. Wasserman, Sudhir Gupta, Paz Carrasco, Jie Chen, Enrique Garcia, Andras Nagy, Leman Yel

**Affiliations:** 1 Albert Einstein College of Medicine and Montefiore Hospital, Bronx, NY USA; 2 Allergy Immunology Medical Center, Redlands, CA USA; 3 Optimed Research, Columbus, OH USA; 4 Duke University, Durham, NC USA; 5 Allergy Partners of North Texas Research, Dallas, TX USA; 6 University of California at Irvine, Irvine, CA USA; 7 Baxalta Innovations GmbH, a Takeda Company, Vienna, Austria; 8 Takeda Development Center Americas, Inc., Cambridge, MA USA

**Keywords:** Immunogenicity, Immunoglobulin replacement, Inborn errors of immunity, Quality of life, Tolerability

## Abstract

Facilitated subcutaneous immunoglobulin (fSCIG) 10% is an immunoglobulin replacement therapy that utilizes recombinant human hyaluronidase (rHuPH20) to enhance immunoglobulin dispersion and absorption, allowing for longer treatment intervals similar to intravenous immunoglobulin (up to once monthly). fSCIG 10% is indicated in the USA for treating adults and children aged ≥ 2 years with primary immunodeficiency diseases (PIDs). This prospective, non-interventional, open-label, multicenter, post-authorization safety study (NCT02593188) was conducted in the USA from November 2015 to October 2021 to assess the long-term safety of fSCIG 10% in routine clinical practice. Patients with PIDs aged ≥ 16 years who were prescribed and/or had started fSCIG 10% treatment were enrolled. In total, 253 patients were enrolled and included (full analysis set). Participants received fSCIG 10% treatment for a median (interquartile range) of 10.0 (3.5–11.8) months, with the majority of infusions administered every 4 weeks (54.4% [1197/2201 infusions]) and at home (62.6% [1395/2230 infusions]). Overall, 98.5% of infusions were administered without rate reduction, interruption, or discontinuation due to adverse events (AEs). Treatment-related, non-serious AEs were experienced by 52 patients (20.6%, 284 events). Two patients (0.8%) each experienced one treatment-related serious AE (aseptic meningitis and deep vein thrombosis). Development of antibodies against rHuPH20 was uncommon; 14/196 patients (7.1%) tested positive for binding antibodies (titer ≥ 1:160) with no neutralizing antibodies detected. There was no relationship between anti-rHuPH20 antibody positivity and the occurrence of treatment-related serious or non-serious AEs. Long-term, repeated self-administration of fSCIG 10% was well tolerated in US clinical practice by patients with PIDs.

**Supplementary Information:**

The online version contains supplementary material available at 10.1007/s10875-024-01769-8.

## Introduction

Facilitated subcutaneous immunoglobulin 10% (fSCIG 10%; HYQVIA [Baxalta US Inc., a Takeda company, Cambridge, MA, USA]) is an immunoglobulin (Ig) replacement therapy that utilizes recombinant human hyaluronidase (rHuPH20). The rHuPH20 component depolymerizes hyaluronan in subcutaneous tissue, transiently increasing tissue permeability and allowing larger volumes of IgG to be administered and absorbed [1]. The associated bioavailability of fSCIG 10% is higher than conventional subcutaneous Ig (SCIG) and comparable to intravenous Ig (IVIG) [2].

fSCIG 10% is approved in the USA for the treatment of adults and children aged 2 years and older with primary immunodeficiency diseases (PIDs; also referred to as inborn errors of immunity) [3]. Like conventional SCIG, fSCIG 10% has fewer systemic adverse reactions than IVIG and can be self-administered at home. In addition, fSCIG 10% can be administered monthly and requires fewer infusions and shorter (cumulative) monthly infusion durations compared with conventional SCIG [4–9].

Previous clinical trials have shown fSCIG 10% to be well tolerated among patients with PIDs [2, 9, 10]. This post-authorization safety study was conducted to assess the long-term safety of fSCIG 10% in adults with PIDs when used in routine clinical practice in the USA. Additional assessments of the safety and tolerability of fSCIG 10% and formation of anti-rHuPH20 antibodies in this population were conducted, as well as evaluation of the effect of fSCIG 10% on several patient-reported treatment satisfaction and health-related quality of life (HRQoL) outcomes.

## Methods

### Study Design

**Fig. 1:**
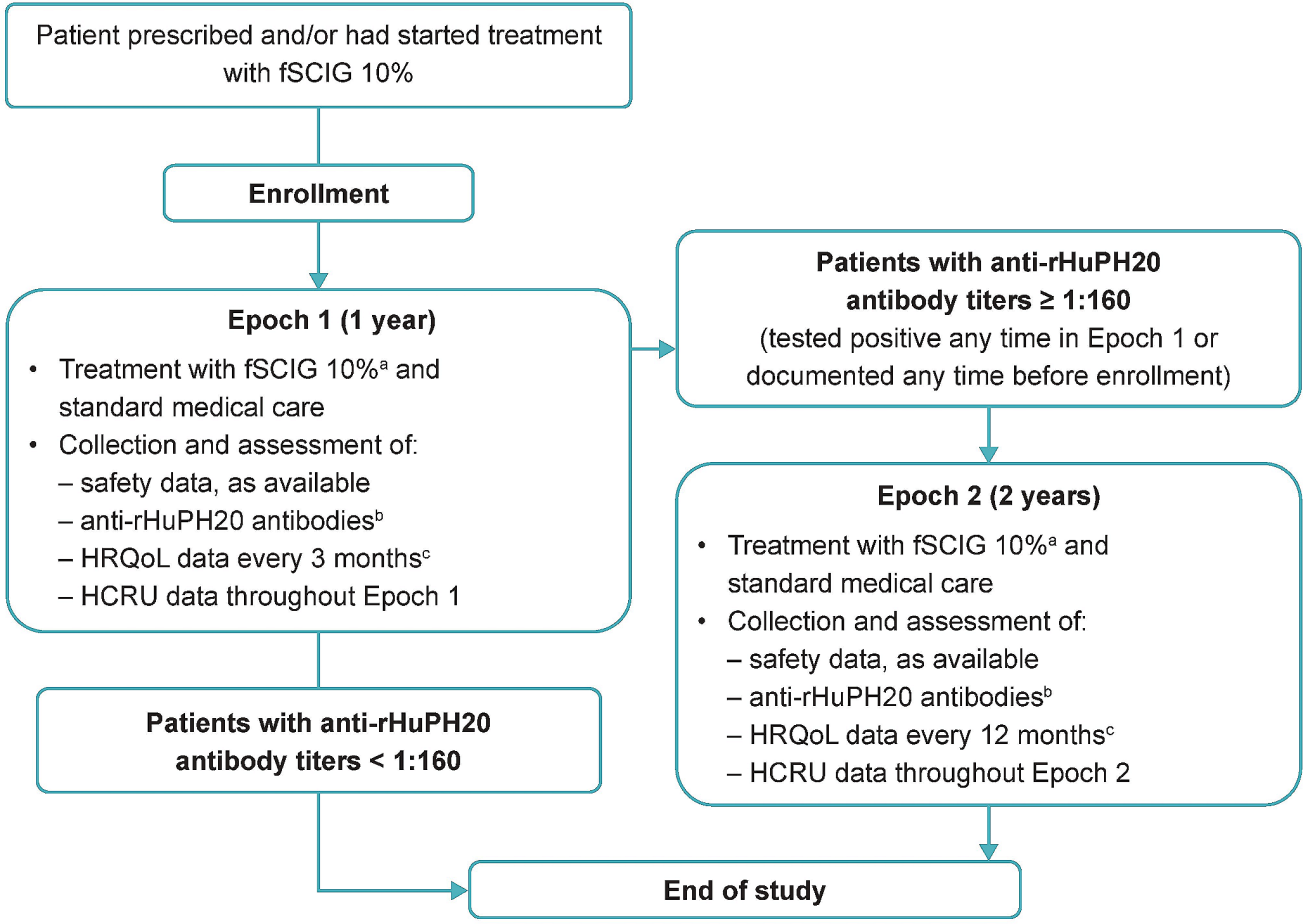
Study design. ^**a**^For patients who discontinued fSCIG 10% at any time during Epoch 1 or Epoch 2, only data on adverse events and assessment of anti-rHuPH20 antibodies at the time of routine laboratory assessments were collected. ^**b**^At the same time as routine laboratory assessments. ^**c**^Patient-reported treatment satisfaction and HRQoL were assessed using questionnaires completed by patients at the screening/enrollment visit, approximately every 3 months during the first year of the study, then annually thereafter, and at the study termination visit (assessed using the Short Form-36 questionnaire version 2, EuroQoL 3-level 5-dimension questionnaire, Treatment Satisfaction Questionnaire for Medication-9, and treatment preference questionnaire). However, the treatment preference questionnaire was collected annually. *fSCIG*, facilitated subcutaneous immunoglobulin; *HCRU*, healthcare resource utilization; *HRQoL*, health-related quality of life; *rHuPH20*, recombinant human hyaluronidase

This prospective, non-interventional, open-label, multicenter, post-authorization safety study (NCT02593188) was conducted in the USA from November 2, 2015 to October 21, 2021. The study comprised two periods, or epochs. In Epoch 1, participants were treated with fSCIG 10% for approximately 1 year; if participants had a positive anti-rHuPH20 antibody titer (≥ 1:160) during this time, or at any time before enrollment, they could enter Epoch 2, during which they received fSCIG 10% treatment for an additional 2 years (Fig. 1). Adverse event (AE) data were collected following enrollment to study completion or discontinuation, and the presence of anti-rHuPH20 antibodies was evaluated on a voluntary basis. If fSCIG 10% was permanently discontinued in Epoch 1, the participant was asked to remain in the study for anti-rHuPH20 antibody and safety assessments.

Patients who required Ig treatment for PID, were aged 16 years and above, were prescribed and/or had started treatment with fSCIG 10%, and were willing and able to comply with the requirements of the protocol, were eligible for enrollment. Treatment regimens were planned by the attending physician in accordance with routine clinical practice. Site visits and all other medical care were performed as standard for the site and for patient healthcare, except for the assessment of anti-rHuPH20 antibodies.

### Study Endpoints

#### Safety

Safety data regarding AEs and serious adverse events (SAEs) were retrieved from participant medical records and associated documentation throughout the study, as well as from voluntary paper or electronic participant diaries that were offered at enrollment for completion during the study. The incidence of total and treatment-related SAEs was recorded, together with the incidence of treatment-related and non-treatment related non-serious AEs.

#### Treatment Regimen and Infusion Interval

Treatment data were retrieved from participant medical records. Details of dose and other infusion parameters were also recorded.

#### Anti-rHuPH20 Antibodies

For the assessment of rHuPH20 binding antibodies, participants were invited to have additional blood samples drawn at the time of routine laboratory assessments approximately every 3 months but not more often than four times a year. For participants with an anti-rHuPH20 antibody titer of 1:160 and above, neutralizing antibodies were measured. Further characterization of antibodies was performed in participants who tested positive for antibodies to rHuPH20 with titers of 1:10 000 and above.

#### Patient-Reported Treatment Satisfaction and Health-Related Quality of Life

Patient-reported treatment satisfaction and HRQoL were assessed using questionnaires completed by patients at the screening/enrollment visit, approximately every 3 months during the first year of the study, then annually thereafter and at the study termination visit. However, patient-reported treatment preference was assessed by questionnaire annually. The Treatment Satisfaction Questionnaire for Medication-9 (TSQM-9) was completed, with three domain scores (effectiveness, convenience, and global satisfaction) recorded. Higher scores indicate greater satisfaction with respect to that domain [11]. A self-administered, non-validated treatment preference questionnaire was used to assess patient preference for various attributes of therapy, including ease of administration, frequency and duration of administration, and convenience. The EuroQoL 5-dimension 3-level (EQ-5D-3L) visual analog scale (VAS) score of the current HRQoL state and index score were evaluated, with higher scores representing better health status, and change from baseline at Month 12 and at the completion visit measured [12]. The Short Form-36 questionnaire version 2 (SF-36v2) physical and mental health component summary scores were assessed, with higher scores indicating better HRQoL, along with change from baseline at Month 12 and at the completion visit [13]. Completion of HRQoL questionnaires was optional.

#### Healthcare Resource Utilization

Healthcare resource utilization (HCRU) assessments, which included hospitalizations, duration of inpatient stays, acute care visits, emergency room visits, and days missed/worked from work/school, were performed at each study visit and at study termination site visits. Completion of HCRU assessments was optional.

### Statistical Analysis

The study was planned to enroll 250 participants with an assumption that all participants would complete Epoch 1. It was estimated that up to 50 patients may test positive for binding rHuPH20 antibodies at a titer of 1:160 and above during Epoch 1 and would therefore become eligible to enter and continue fSCIG 10% treatment in Epoch 2 [10]. All patients who provided informed consent and met enrollment eligibility were included in the full analysis set. The full analysis set was the main analysis population in this study.

Statistical data were descriptive. Categorical variables were summarized using frequencies and percentages, and continuous variables were summarized using mean, standard deviation (SD), median, 25th percentile (Q1), 75th percentile (Q3), minimum, and maximum, as appropriate. All data processing and summarization were conducted using SAS^®^, version 9.4. Missing data were not imputed, and data were evaluated and presented as recorded in the study database. Missing items on patient-reported questionnaires were handled according to questionnaire-scoring guidelines for missing data.

## Results

### Participant Demographics and Baseline Characteristics

**Fig. 2:**
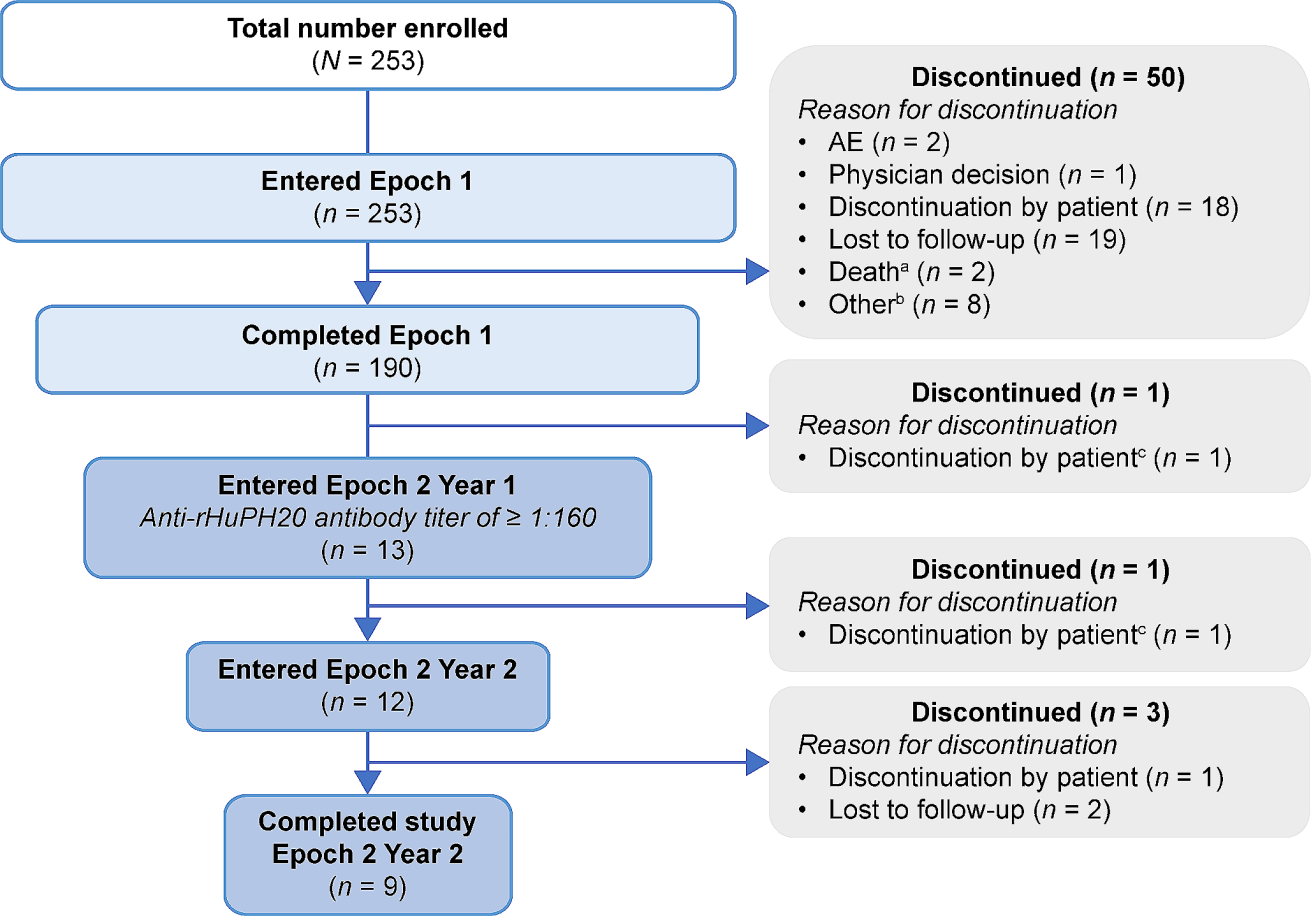
Patient disposition. ^**a**^Two fatal AEs (possible stress-related complication of chronic lymphocytic leukemia; cardiogenic shock) were reported, neither of which was considered to be treatment-related. ^**b**^Other reasons included: unable to keep timely appointments (*n =* 2); switched to home infusion and would not complete diary and/or questionnaire (*n =* 2); switched to home infusion (*n =* 1); switched to another treatment (*n =* 1); transferred care (*n =* 1); did not restart fSCIG 10% (*n =* 1). ^**c**^Participant eligible for entry into Epoch 2 (anti-rHuPH20 antibody titer of ≥ 1:160 in Epoch 1) but withdrew consent before entry. *AE* adverse event, *fSCIG* facilitated subcutaneous immunoglobulin, *rHuPH20* recombinant human hyaluronidase

In total, 253 patients were enrolled into the study and were included in the full analysis set (Fig. 2). Two out of the 50 participants who discontinued the study during Epoch 1 withdrew consent because of AEs. One patient experienced fatigue, which was of moderate severity, probably related to treatment and later resolved/recovered. The AEs experienced by the second patient included pyrexia, headache, and myalgia, all of which were mild, possibly related to treatment, and later resolved/recovered. One of these patients was negative for binding anti-rHuPH20 antibodies and the other patient did not undergo testing. No participants discontinued the study during Epoch 2 owing to AEs. None of the enrolled patients tested positive for binding anti-rHuPH20 antibodies at any time prior to enrollment. Among 14 participants who were positive for treatment-emergent anti-rHuPH20-binding antibodies during Epoch 1, 13 entered Epoch 2 (1 patient withdrew consent). Most participants in the study had a history of Ig treatment (*n =* 242/253, 95.7%) and many patients had ongoing fSCIG 10% treatment at enrollment (*n =* 141/169, 83.4%). Participant demographics and characteristics are summarized in Table 1.

**Table 1:** Baseline participant characteristics

Characteristic	Full analysis set (*N =* 253)
Age at informed consent, years	
Mean (SD)	54.3 (15.6)
Median (IQR)	57.0 (44.0–66.0)
Age category, years, *n* (%)	
16 to < 30	23 (9.1)
30 to < 40	23 (9.1)
40 to < 50	39 (15.4)
50 to < 60	63 (24.9)
60 to < 65	28 (11.1)
≥ 65	77 (30.4)
Sex, *n* (%)	
Female	200 (79.1)
Male	53 (20.9)
Race, *n* (%)	
White	234 (92.5)
Unknown/not available	10 (4.0)
American Indian or Alaskan Native	5 (2.0)
Black/African American	3 (1.2)
Asian	1 (0.4)
Ethnicity, *n* (%)	
Non-Hispanic or Latino	240 (94.9)
Hispanic or Latino	11 (4.3)
Not applicable	2 (0.8)
BMI category, kg/m^2^, *n*^a^	
< 18.5	7
18.5 to < 25.0	61
25.0 to < 30.0	69
≥ 30.0	89
PID type, *n* (%)	
Common variable immunodeficiency	182 (71.9)
Hypogammaglobulinemia NOS	38 (15.0)
Specific antibody deficiency	22 (8.7)
Agammaglobulinemia NOS	3 (1.2)
IgG subclass deficiency	3 (1.2)
Selective IgM deficiency	2 (0.8)
CD4 lymphocytopenia	1 (0.4)
X-linked agammaglobulinemia	1 (0.4)
Unknown^b^	1 (0.4)

^a^Among participants with BMI data (*n =* 226)

^b^No recorded PID type for participant

*BMI* body mass index, *CD4* cluster of differentiation 4, *IgG* immunoglobulin G, *IgM* immunoglobulin M, *IQR* interquartile range, *NOS* not otherwise specified, *PID* primary immunodeficiency disease, *SD* standard deviation

### Treatment Characteristics

Participants received fSCIG 10% treatment for a median (interquartile range [IQR]) duration of 10.0 (3.5–11.8) months. Participants who discontinued fSCIG 10% could remain in the study for anti-rHuPH20 antibody and safety assessments. Among the 37 participants who discontinued fSCIG 10% permanently at an Epoch 1 visit, 14 participants remained in the study. During Epoch 2, two participants discontinued fSCIG 10% and 21 participants discontinued fSCIG 10% at a completion/termination visit. fSCIG 10% discontinuation due to AEs occurred in four, one, and six participant(s) at Epoch 1, Epoch 2, and the completion/termination visits, respectively.

Infusion parameters for participants with available treatment data during follow-up are summarized in Table 2. The majority of infusions were administered every 4 weeks (1197/2201, 54.4%, *n =* 225) and at home (1395/2230, 62.6%, *n =* 227), with a median (IQR) number of infusion sites of 2 (2.0–2.0, *n* = 203). Of participants with available data (*n =* 227), most (*n =* 144, 63.4%) used two sites, 30 (13.2%) used only a single site, and 29 (12.8%) used one or two sites. The most frequently used infusion site location was the abdomen, including the left upper abdomen (22.5%), right upper abdomen (22.3%), right lower abdomen (18.1%), and left lower abdomen (17.0%).

**Table 2:** fSCIG 10% treatment characteristics

Characteristic	Full analysis set (*N =* 253)
Location of infusion^a^, *n* (%)	
Home	1395 (62.6)
Clinical site	835 (37.4)
Frequency of administration^b^, *n* (%)	
1 week	21 (1.0)
2 weeks	297 (13.5)
3 weeks	478 (21.7)
4 weeks	1197 (54.4)
> 4 weeks	161 (7.3)
Other^c^	47 (2.1)
Infusion duration, hours	
Participants^b^, *n*	148
Median (IQR)	3.0 (2.0–4.0)
Infusion sites, *n*	
Participants^b^, *n*	203
Median (IQR)	2.0 (2.0–2.0)
Mean (SD)	1.9 (0.5)
Administered volume of IgG, mL	
Participants^b^, *n*	201
Median (IQR)	400.0 (300.0–425.0)
Maximum Ig infusion rate, mL/hour	
Participants^b^, *n*	189
Median (IQR)	240.0 (212.0–300.0)

^a^Number of treatments during the study period with non-missing data

^b^Number of patients with non-missing data for the infusion parameter

^c^A specific number of days that did not fall into other administration categories

*fSCIG* facilitated subcutaneous immunoglobulin, *Ig* immunoglobulin, *IgG* immunoglobulin G, *IQR* interquartile range, *SD* standard deviation

### Safety and Tolerability

Overall, 98.5% of infusions were administered without rate reduction, interruption, or discontinuation due to AEs. Treatment-related, non-serious AEs (including infections) were experienced by 52 participants (20.6%, 284 events) (Table 3). Overall, the rate of all SAEs was 0.207 per person-year (95% confidence interval [CI]: 0.159–0.266). The incidences of serious, non-serious, and local and systemic AEs related to fSCIG 10% are shown in Table 4. Most treatment-related AEs were either mild or moderate in severity. Two participants (0.8%) each experienced one treatment-related SAE (aseptic meningitis and deep vein thrombosis). Two fatal AEs (possible stress-related complication of chronic lymphocytic leukemia, and cardiogenic shock; 0.8%) were reported, neither of which was considered to be treatment-related. The incidence of all non-serious AEs (number of events per person-year, including infections) was lower among patients previously treated with fSCIG 10% (3.068, 532 events, *n =* 98/141) than those naive to fSCIG 10% treatment at enrollment (5.790, 161 events, *n =* 21/28). There was also a marked difference in the number of events per person-year of systemic non-serious AEs between participants previously treated with fSCIG 10% (0.623, 108 events, *n =* 21/141) and those naive to fSCIG 10% treatment (1.690, 47 events, *n =* 5/28).

**Table 3:** Treatment-related AEs, including infections

Event	Participants, *n*		Events, *n*	Events per participant, *n*	Events per infusion, *n*
Total non-serious AEs^a^	52	284		1.123	0.127
Local AEs	28	98		0.387	0.044
Systemic AEs	36	185		0.731	0.083
Serious AEs	2	2		0.008	0.001
Systemic AEs^b^	2	2		0.008	0.001

^a^One non-serious AE is missing information and not categorized as a local/systemic AE

^b^No local serious AEs were recorded

*AE* adverse event

**Table 4:** Treatment-related AEs by severity and by preferred term

	Participants, *n*	Events, *n*	Events per participant, *n*	Events per infusion, *n*
**AE by severity, including infections**				
Mild Moderate Severe	40 22 4	153 126 7	0.605 0.498 0.028	0.068 0.056 0.003
**AE by severity**,** excluding infections**				
Mild Moderate Severe	40 22 3	153 126 6	0.605 0.498 0.024	0.068 0.056 0.003
**AE by preferred term**^**a **^**and severity**,** including infections**				
Headache				
Mild Moderate Severe	7 7 1	12 24 1	0.047 0.095 0.004	0.005 0.011 < 0.001
Infusion site pain^b^				
Mild Moderate	10 3	20 4	0.079 0.016	0.009 0.002
Infusion site swelling^b^				
Mild Moderate	7 4	8 15	0.032 0.059	0.004 0.007
Fatigue				
Mild Moderate Severe	6 3 1	10 7 3	0.040 0.028 0.012	0.004 0.003 0.001

^a^Listed if occurring in ≥ 2% of patients

^b^No severe AEs were reported

*AE* adverse event

### Immunogenicity

Of 196 participants enrolled, for whom at least one anti-rHuPH20 assessment was completed, 14 (7.1%) tested positive for treatment-emergent anti-rHuPH20-binding antibodies (maximum titer 1:10 240). Samples from four patients with anti-rHuPH20 antibody titers ≥ 1:10 000 did not cross-react with other human hyaluronidases (hyaluronidase-1, hyaluronidase-2, and hyaluronidase-4). The incidence of a positive anti-rHuPH20 antibody test according to the specific PID diagnosis was highest in participants with specific antibody deficiency (11.8%; 95% CI: 3.3–34.3) followed by hypogammaglobulinemia (9.1%; 95% CI: 3.1–23.6) and common variable immunodeficiency (6.6%; 95% CI: 3.5–12.0). Among 150 participants with an assessment at baseline, eight (5.3%) were positive for anti-rHuPH20 antibodies, six (75.0%) of whom were treated with fSCIG 10% prior to enrollment.

**Fig. 3:**
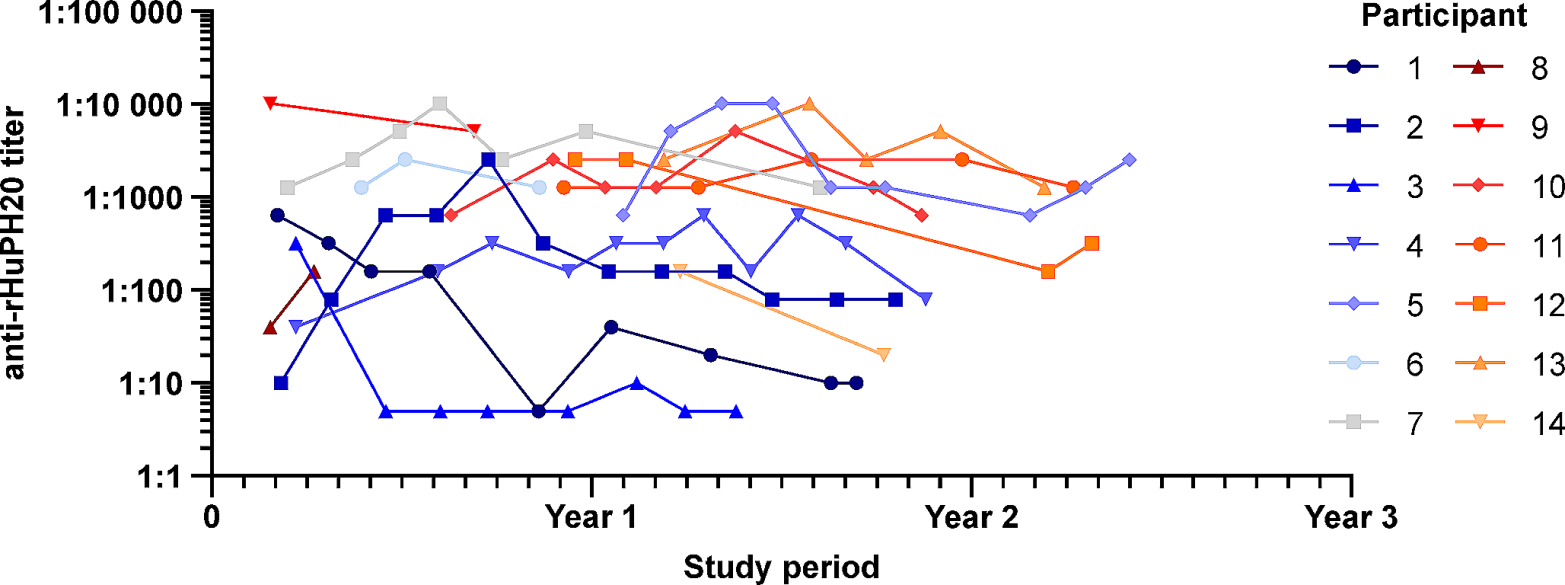
Anti-rHuPH20 antibody titers over Epochs 1 and 2. *rHuPH20*, recombinant human hyaluronidase

Overall, among participants with positive antibody titers, titers generally increased over Epoch 1, with varying trends over time in Epoch 2 (Fig. 3). However, the incidence of non-serious AEs related to fSCIG 10% treatment did not increase after the first positive anti-rHuPH20 titer. No participants experienced any treatment-related SAEs before or after the first positive antibody test and no neutralizing antibodies were detected.

### Patient-Reported Outcomes

Most respondents reported self-administration to be easy (22/56, 39.3%) or very easy (18/56, 32.1%) at Month 12 of Epoch 1 (Supplementary Table S1). During Epoch 1, among respondents with available treatment preference data (*n =* 91), almost all (98.9%) planned to continue fSCIG 10% treatment.

In general, HRQoL measurements remained stable over the study. The mean (SD) change in the EQ-5D-3L VAS score from baseline to Month 12 of Epoch 1 was 2.5 (15.88) (Supplementary Table S2). The EQ-5D-3L questionnaire results are summarized in Supplementary Table S3. The proportion of respondents who reported some anxiety/depression or problems with activities decreased over Epoch 1 (33.6% at baseline to 23.9% at Month 12 of Epoch 1) (Supplementary Table 31). By contrast, the proportion of respondents reporting some problems with pain/discomfort increased over Epoch 1 (54.1% at baseline to 62.7% at Month 12 of Epoch 1).

SF-36v2 scores were indicative of normal health and remained stable over Epoch 1 (mean [SD] changes from baseline to Month 12 of Epoch 1 were − 0.4 [5.49] and 0.5 [8.16] for physical and mental component summary scores, respectively) (Supplementary Table S4).

### Healthcare Resource Utilization

At the time of study completion/discontinuation, 56 hospitalization events (event rate [number of events per person-year]: 0.191) and 287 days in hospital (event rate: 0.980) had occurred. In total, 15 participants (event rate [number of events per person-year]: 0.051) had at least one hospitalization event owing to an infection (Supplementary Table S5). There were no abnormal total IgG levels within the 4 weeks prior to or during hospitalization.

## Discussion

The results of this study show that long-term, repeated self-administration of fSCIG 10% was well tolerated when used to treat patients with PID in routine US clinical practice. Over a median (IQR) exposure period of 10.0 (3.5–11.8) months, the most common dosing interval used for fSCIG 10% was every 4 weeks with a mean (SD) number of 1.9 (0.53) infusion sites. This dosing interval aligns with that recommended in the US prescribing information [3]. The number of infusion sites reported here was higher than in a similar post-authorization safety study conducted in the EU (1.1 [0.4]) [14]. This observation indicates that there may be differences in clinical practice between physicians in the USA and EU regarding the number of infusion sites used.

Overall, the incidence of all SAEs was 0.21 per person-year (95% CI: 0.16–0.27), which is in accordance with data from the EU post-authorization safety study (0.27 per person-year) [14]. However, this rate is higher than that reported in the pivotal study of fSCIG 10% among patients aged 18 years and above (0.13 per person-year), which may be attributed to differences in observation time and the high proportion of elderly participants included in this study. In the current study, 28 (11.1%) participants were aged between 60 years and under 65 years, with 77 (30.4%) aged 65 years and older. Overall, systemic AEs occurred more frequently than local AEs. The proportion of elderly participants in this study and the potential for selection bias for patients experiencing AEs in a safety study could explain this finding.

Development of positive titers against rHuPH20 was uncommon (*n =* 14 [7.1%]), with no neutralizing antibodies detected. The prevalence of anti-rHuPH20 antibodies reported here is consistent with the range of reported rates from clinical trials of various subcutaneous treatments co-administered with rHuPH20 (0.9–44.7%) [15, 16]. It is also important to consider that the prevalence of antibodies reactive to rHuPH20 in healthy donors in the absence of exposure to rHuPH20 ranges between 1.6% and 12.1% [15, 17]. In addition, data from the current study align with the EU post-authorization safety study and indicate a lower incidence of positive titers for binding rHuPH20 antibodies than in the integrated analysis of the pivotal study and its extension [10, 14]. However, in the pivotal trial and its extension, only patients naive to fSCIG 10% were enrolled and the final data showed that anti-rHuPH20 antibody titers typically declined during treatment [10]. In this study, most participants with positive titers were treated with fSCIG 10% before study enrollment. Overall, in investigations of the potential immunogenicity of rHuPH20, no clinical signs or symptoms have been associated with a positive rHuPH20 antibody response and no rHuPH20-reactive antibodies capable of neutralizing hyaluronidase activity have been confirmed to date in patients with PID. The lack of clinical significance associated with rHuPH20-reactive antibodies may be due to the local and transient effect of rHuPH20 in the subcutaneous space, and the very restricted expression pattern of the endogenous enzyme [18].

In general, mean HRQoL measurements remained stable over the study. Overall, low rates of HCRU events were observed, with no hospitalizations in Year 1 of Epoch 2. In addition, mean TSQM-9 scores were generally high throughout the study for treatment satisfaction domains pertaining to fSCIG 10% effectiveness, convenience, and global satisfaction.

There are a few limitations of our study that should be highlighted. Despite several steps taken to limit the effect of bias and confounding in the study, limitations of the observational design and conduct should be noted when interpreting the results. For example, selection bias, potentially induced from eligibility screening coinciding with a participant’s regular visit or a treatment-related visit at the treatment center, should be considered. In addition, follow-up bias may have occurred because several participants (8.3%) were lost to follow-up during the study, coinciding with the COVID-19 pandemic. The study also relied on voluntary anti-rHuPH20 antibody assessments and participant self-reported outcomes, which can further contribute to potential bias. Another limitation to consider is that fSCIG 10% was relatively new to the US market when the study started and, therefore, the characteristics of patients initiating treatment at the very beginning of the study may have differed from those of patients starting later. Most of the participants were treated with SCIG before enrollment and therefore the occurrence of local AEs observed in this study may differ from that of participants who were treatment-naive or had newly initiated fSCIG 10% treatment, given that local AEs generally decrease over time [9]. Selective prescribing of a specific medication to patients with a different clinical profile, such as those with more severe PID, could have occurred. Finally, the sample was affected, in part, by missing data, as is expected in real-world research; for example, 62.6% of the participants self-administered fSCIG 10% at home during the study, and it is likely that they missed reporting some infusion and AE data. Nonetheless, the large sample size of 253 participants observed over a median follow-up period of 10 months improves confidence in the data presented.

## Conclusions

The results of this study show that long-term repeated self-administration of fSCIG 10% was well tolerated in patients with PID in routine US clinical practice. Development of positive titers against rHuPH20 was uncommon and did not correlate with the occurrence of treatment-related AEs. This study provides valuable insight into fSCIG 10% treatment and product administration in the USA and confirms clinical practice differences in dosing compared with the EU, further highlighting the flexibility of product administration.

## Electronic Supplementary Material

Below is the link to the electronic supplementary material.

Supplementary Material 1

## Data Availability

The data sets, including the redacted study protocol, redacted statistical analysis plan, and individual participant data supporting the results reported in this article, will be made available within 3 months of initial request, to researchers who provide a methodologically sound proposal. The data will be provided after their de-identification, in compliance with applicable privacy laws, data protection, and requirements for consent and anonymization.
