# Supplementary material for: Long-Term Safety of Facilitated Subcutaneous Immunoglobulin 10% Treatment in US Clinical Practice in Patients with Primary Immunodeficiency Diseases: Results from a Post-Authorization Safety Study
Source: J Clin Immunol. 2024 Aug 19;44(8):181. doi: 10.1007/s10875-024-01769-8 (PMC11333520; doi:10.1007/s10875-024-01769-8)
Supplement: Supplementary file 1 — Supplementary Material 1 [file 10875_2024_1769_MOESM1_ESM.pdf]

## Supplementary material

**Supplementary Table S1** Summary of patient-reported satisfaction as measured by the Treatment Satisfaction Questionnaire for Medication-9 at Epoch 1 Month 12

| Parameter | Full analysis set (*N* = 253) | |
|---|---|---|
| | Observed values | Change from baseline[a] |
| **Effectiveness** | | |
| *n* | 71 | 51 |
| Mean (SD) | 72.0 (22.93) | 5.5 (21.16) |
| Median (IQR) | 72.2 (61.1, 88.9) | 0.0 (−5.6, 16.7) |
| Missing, *n* | 0 | 20 |
| **Convenience** | | |
| *n* | 71 | 52 |
| Mean (SD) | 68.8 (17.90) | 3.8 (19.64) |
| Median (IQR) | 66.7 (55.6, 83.3) | 0.0 (−5.6, 13.9) |
| Missing, *n* | 0 | 19 |
| **Satisfaction** | | |
| *n* | 71 | 52 |
| Mean (SD) | 77.3 (19.70) | 2.6 (18.66) |
| Median (IQR) | 78.6 (64.3, 92.9) | 0.0 (−7.1, 10.7) |
| Missing, *n* | 0 | 19 |

[a]Baseline is defined as the non-missing measurement taken at enrollment.
If multiple visits are available within the same visit window, the data collected closest to the mid-point are used. If two observations are of equal distance to the mid-point, the most recent observation is used.
Data from nine participants were not included in the analysis owing to incomplete casebook documentation.
IQR, interquartile range; SD, standard deviation.

**Supplementary Table S2** Summary of health-related quality of life as measured by the EuroQoL 5-dimension 3-level visual analog scale score at baseline and Epoch 1 Month 12

| **Parameter** | **Full analysis set (*N* = 253)** | |
|---|---|---|
| | **Observed value** | **Change from baseline[a]** |
| **Epoch 1 Month 12** | | |
| *n* | 67 | 50 |
| Mean (SD) | 73.1 (16.84) | 2.5 (15.88) |
| Median (IQR) | 75.0 (66.0, 85.0) | 0.5 (−10.0, 10.0) |
| Missing, *n* | 25, 98 | −26, 50 |

[a]Baseline is defined as the non-missing measurement taken at enrollment.
If multiple visits are available within the same visit window, the data collected closest to the mid-point are used. If two observations are of equal distance to the mid-point, the most recent observation is used.
Data from nine participants were not included in the analysis owing to incomplete casebook documentation.
IQR, interquartile range; SD, standard deviation.

**Supplementary Table S3** Summary of health-related quality of life as measured by the EuroQoL 5-dimension 3-level questionnaire at baseline and Epoch 1 Month 12

| **Parameter** | **Full analysis set (*N* = 253)** | | | | |
|---|---|---|---|---|---|
| | **Mobility** | **Self-care** | **Activities** | **Pain/discomfort** | **Anxiety/ depression** |
| **Baseline, *n* (%)** | | | | | |
| *n* | 146 | 146 | 146 | 146 | 146 |
| No problem | 94 (64.4) | 128 (87.7) | 69 (47.3) | 49 (33.6) | 89 (61.0) |
| Some problem | 52 (35.6) | 17 (11.6) | 72 (49.3) | 79 (54.1) | 49 (33.6) |
| Extra problem | 0 (0.0) | 1 (0.7) | 5 (3.4) | 18 (12.3) | 8 (5.5) |
| **Epoch 1 Month 12, *n* (%)** | | | | | |
| *n* | 67 | 67 | 67 | 67 | 67 |
| No problem | 41 (61.2) | 57 (85.1) | 36 (53.7) | 22 (32.8) | 49 (73.1) |
| Some problem | 26 (38.8) | 10 (14.9) | 29 (43.3) | 42 (62.7) | 16 (23.9) |
| Extra problem | 0 (0.0) | 0 (0.0) | 2 (3.0) | 3 (4.5) | 2 (3.0) |

If multiple visits are available within the same visit window, the data collected closest to the mid-point are used. If two observations are of equal distance to the mid-point, the most recent observation is used.
Data from nine participants were not included in the analysis owing to incomplete casebook documentation.

**Supplementary Table S4** Summary of health-related quality of life as measured by the Short Form-36 questionnaire version 2 at Epoch 1 Month 12

| Parameter | Full analysis set (*N* = 253) | |
|---|---|---|
| | **Observed value** | **Change from baseline**[a] |
| **PCS score** | | |
| *n* | 76 | 61 |
| Mean (SD) | 39.2 (11.26) | −0.4 (5.49) |
| Median (IQR) | 39.5 (29.7, 47.7) | −0.3 (−3.0, 3.2) |
| **MCS score** | | |
| *n* | 76 | 61 |
| Mean (SD) | 49.4 (9.80) | 0.5 (8.16) |
| Median (IQR) | 50.3 (44.0, 57.3) | 0.9 (−3.9, 4.5) |
| **Physical functioning** | | |
| *n* | 76 | 61 |
| Mean (SD) | 62.4 (31.29) | −2.8 (15.52) |
| Median (IQR) | 65.0 (37.5, 95.0) | 0.0 (−5.0, 2.4) |
| **Role-physical** | | |
| *n* | 76 | 61 |
| Mean (SD) | 56.4 (32.14) | −2.3 (19.12) |
| Median (IQR) | 56.3 (25.0, 87.5) | −6.3 (−12.5, 6.3) |
| **Bodily pain** | | |
| *n* | 76 | 61 |
| Mean (SD) | 53.1 (27.17) | 1.5 (22.63) |
| Median (IQR) | 46.0 (31.5, 74.0) | 0.0 (−10.0, 12.0) |
| **General health** | | |
| *n* | 76 | 61 |
| Mean (SD) | 40.2 (20.19) | 2.3 (15.24) |
| Median (IQR) | 37.0 (27.0, 49.5) | 0.0 (−5.0, 10.0) |
| **Vitality** | | |
| *n* | 76 | 61 |
| Mean (SD) | 44.6 (24.54) | 0.7 (20.06) |
| Median (IQR) | 50.0 (25.0, 62.5) | 0.0 (−6.3, 8.3) |
| **Social functioning** | | |
| *n* | 76 | 61 |

| | | |
|---|---|---|
| Mean (SD) | 64.3 (24.89) | −1.6 (19.43) |
| Median (IQR) | 62.5 (50.0, 87.5) | 0.0 (−12.5, 12.5) |
| **Role-emotional** | | |
| *n* | 76 | 61 |
| Mean (SD) | 78.2 (29.12) | −1.0 (24.58) |
| Median (IQR) | 95.8 (66.7, 100.0) | 0.0 (−8.3, 8.3) |
| **Mental health** | | |
| *n* | 76 | 61 |
| Mean (SD) | 75.3 (17.72) | 2.1 (12.30) |
| Median (IQR) | 80.0 (67.5, 90.0) | 0.0 (−5.0, 10.0) |

[a]Baseline is defined as the non-missing measurement taken at enrollment.
If multiple visits are available within the same visit window, the data collected closest to the mid-point are used. If two observations are of equal distance to the mid-point, the most recent observation is used.
Data from nine participants were not included in the analysis owing to incomplete casebook documentation.
IQR, interquartile range; MCS, mental health component summary; PCS, physical health component summary; SD, standard deviation.

**Supplementary Table S5** Infection-related healthcare resource use over time during study

| | Full analysis set (*N* = 253) | | | |
|---|---|---|---|---|
| **Healthcare resource use** | ***N*** | **Person-years** | **Event rate[b]** | **95% CI for event rate** |
| Patients with ≥ 1 hospitalization event | 15 | 292.88 | 0.051 | 0.029, 0.084 |
| Hospitalization event[a] | 21 | 292.88 | 0.072 | 0.044, 0.110 |
| Days in hospital[a] | 135 | 292.88 | 0.461 | 0.386, 0.546 |

[a]Multiple events can occur for a single participant.
[b]Event rate = number of events/person-years.
Data from nine participants were not included in the analysis owing to incomplete casebook documentation.
CI, confidence interval.
